# Impact of disease stage and aetiology on survival in hepatocellular carcinoma: implications for surveillance

**DOI:** 10.1038/bjc.2016.422

**Published:** 2017-01-12

**Authors:** Philip Johnson, Sarah Berhane, Chiaki Kagebayashi, Shinji Satomura, Mabel Teng, Richard Fox, Winnie Yeo, Frankie Mo, Paul Lai, Stephen L Chan, Toshifumi Tada, Hidenori Toyoda, Takashi Kumada

**Affiliations:** 1 Department of Molecular and Clinical Cancer Medicine, University of Liverpool and Clatterbridge Cancer Centre NHS Foundation Trust, Sherrington Building, Ashton Street, Liverpool, Merseyside L69 3GA, UK; 2 The Clatterbridge Cancer Centre NHS Foundation Trust, Clatterbridge Road, Bebington, Wirral CH63 4JY, UK; 3 Department of Molecular Biochemistry and Clinical Investigation, Osaka University Graduate School of Medicine, Osaka, Japan; 4 Department of Oncology, Addenbrooke's Hospital, University of Cambridge, Hills Road, Cambridge CB2 0QQ, UK; 5 Cancer Research UK Clinical Trials Unit, School of Cancer Sciences, University of Birmingham, Birmingham B15 2TT, UK; 6 State Key Laboratory in Oncology in South China, Sir YK Pao Centre for Cancer, Department of Clinical Oncology, Chinese University of Hong Kong, Hong Kong Cancer Institute, Hong Kong, China; 7 Department of Surgery, Prince of Wales Hospital, Chinese University of Hong Kong, Hong Kong, China; 8 Department of Gastroenterology and Hepatology, Ogaki Municipal Hospital, 4-86 Minaminokawa-cho, Ogaki, Gifu 503-8052, Japan

**Keywords:** surveillance, geographical variation, hepatocellular carcinoma, hepatitis B virus, aetiology, hepatitis C virus

## Abstract

**Background:**

Variation in survival in hepatocellular carcinoma (HCC) has been attributed to different aetiologies or disease stages at presentation. While international guidelines recommend surveillance of high-risk groups to permit early diagnosis and curative treatment, the evidence that surveillance decreases disease-specific mortality is weak.

**Methods:**

We compared HCC survival figures from Japan (*n*=1174) and Hong Kong (*n*=1675) over similar time periods (Japan 2000–2013, Hong Kong, China 2003–2014). The former has an intensive national surveillance programme, while the latter has none. We also analysed changes in survival in Japan over a 50-year period including data from before and after institution of a national HCC surveillance programme.

**Results:**

In Japan, over 75% of cases are currently detected by surveillance, whereas in Hong Kong <20% of cases are detected presymptomatically. Median survival was 52 months in Japan and 17.8 months in Hong Kong; this survival advantage persisted after allowance for lead-time bias. Sixty-two per cent of Japanese patients had early disease at diagnosis and 63% received curative treatment. The comparable figures for Hong Kong were 31.7% and 44.1%, respectively. These differences could not be accounted for by disease aetiology, and patients in Hong Kong who were detected at an early stage had a similar survival to the analogous patients in Japan.

**Conclusions:**

The variation in survival is largely accounted for by stage at diagnosis, which in turn relates to the intensity of surveillance programmes and the consequent variation in curative therapeutic options.

A striking feature of hepatocellular carcinoma (HCC) is the wide global variation in incidence, ranging from <3/100 000 in Northern Europe to >30/100 000 in parts of Africa and China (Parkin *et al*, 2005). In all regions, most HCC arises in the setting of chronic liver disease (Llovet, 2005). Equally striking is the wide variation in reported median survival figures ranging from <3 months in parts of Africa to >3 years in Taiwan and Japan (Chen *et al*, 2006; Hsu *et al*, 2010).

Recognising the crucial importance of early diagnosis for the implementation of potentially curative therapy, most international guidelines suggest that patients at high HCC risk (Omata *et al*, 2010; Bruix and Sherman, 2011; For Research, EO and Liver, EAFTSOT, 2012; Song *et al*, 2012) are screened by six monthly ultrasound (US) examinations with or without the serum tumour marker *α*-fetoprotein (AFP). However, systematic reviews conclude that the evidence that surveillance decreases disease-specific mortality is weak (Kansagara *et al*, 2014) and the US National Cancer Institute concludes that ‘surveillance of persons at elevated risk does not result in a decrease in mortality from hepatocellular cancer' (NCI). The only randomised trial showing benefit from surveillance (Zhang *et al*, 2004) had significant methodological limitations (Kansagara *et al*, 2014). Furthermore, although those who are detected within a surveillance programme tend to have ‘earlier' disease and survive longer, the possibility that this is attributable to lead-time bias is difficult to exclude (Singal *et al*, 2014; Sherman, 2014b). However, it is recognised that a formal randomised trial of surveillance to provide the relevant evidence base is now impossible, not least because properly informed patients would not consent to recruitment to a control, unscreened arm, particularly in the light of international clinical guidelines (McCaughan, 2013; Kansagara *et al*, 2014; Singal *et al*, 2014; Sherman, 2014b). On the basis of this lack of evidence, most Western countries have chosen not to implement a national surveillance programme and it has been left to individual hospitals or clinicians to undertake surveillance resulting in very variable practice (Dalton-Fitzgerald *et al*, 2014; Joshi *et al*, 2014). Thus, patients are caught between guidelines written by HCC ‘experts' who strongly support surveillance and funders who are reluctant to act on these guidelines in the absence of a firm conventional evidence base, while both sides recognise that such an evidence base is impossible to acquire.

In an attempt to provide some evidence as to the potential benefits of surveillance for HCC without a formal randomised trial, we have compared HCC survival rates in two national patient cohorts both with advanced and sophisticated health-care systems. One of these, Japan, has a mature, intensive, national programme of surveillance for HCC, whereas the second, Hong Kong, has not introduced such a programme.

## Patients and methods

The study involved patient level data from HCC centres in Japan and Hong Kong, China. Part of the Japan cohort has previously been reported by Toyoda *et al* (2006b), whereas the Chinese cohort comprised consecutive patients drawn from the North West Territories, Hong Kong. We also had access to historical data pertaining to changes occurring in the same region of Japan over the period between 1969 and 2013 in terms of age, survival, 90-day postoperative mortality, tumour size and stage (as assessed by the Japanese Integrated Staging (JIS) score) with which to assess the impact of the introduction of surveillance in 1980.

### Diagnosis, tumour characteristics and assessment of survival

Patients were diagnosed on the basis of characteristic radiology according to international guidelines (For Research, EO and Liver, EAFTSOT, 2012) or histological examination of tumour tissue. Survival was calculated from date of diagnosis. Parameters recorded common to both cohorts are shown in Table 1. Aetiology was classified as hepatitis B virus (HBV) or hepatitis C virus (HCV) related or ‘other', the latter including alcoholic and other forms of chronic liver disease (Table 1). Where aetiology was mixed, typically HCV and alcohol, the former was recorded.

### Treatment and staging

Japanese patients were staged according to the JIS score (Kudo *et al*, 2003). In Hong Kong (China), treatment was decided in multidisciplinary meetings. Liver transplantation was not available in the Hong Kong or Japanese centres. Both units had ready access to ‘state-of-the-art' treatments, which was not influenced by cost considerations. The Milan Criteria (three tumours <3 cm or one tumour <5 cm; Mazzaferro *et al*, 1996) was used to classify patients as having early (potentially curative disease) or advanced disease (Singal *et al*, 2014). Resection, radiofrequency ablation and percutaneous ethanol injection were considered potentially curative treatments. All other treatment options were considered palliative.

### Surveillance policy

Mass surveillance was introduced in Japan in 1980. The approach adopted in the Ogaki prefecture, described here, is typical of the whole of Japan (The Japan Society of Hepatology, 2010a, 2010b; Kudo *et al*, 2016). The population above the age of 50 years is offered regular screening for chronic viral hepatitis. All patients with cirrhosis or severe fibrosis are followed-up with US examination every 3–6 months; no patients are excluded on the grounds of advanced liver disease/liver failure. Regular monitoring of tumour markers (AFP, AFP-L3% and des-gamma-carboxy prothrombin) is also performed every 3–6 months. When an increase of tumour markers is observed, additional imaging examinations are performed. In Hong Kong, China there was no formal surveillance programme.

### Statistical methods

Statistical analysis was undertaken using Stata IC 12 (Stata Corp, College Station, TX, USA). Survival curves were generated by the Kaplan–Meier method. Univariable Cox regression analysis was used to identify significant prognostic variables in each of the cohorts. Variables analysed were age, gender, albumin (g l^−1^), AFP (ng ml^−1^), bilirubin (*μ*mol l^−1^), treatment (curative/palliative), tumour size (cm), tumour type (solitary or multifocal), vascular invasion, aetiology (HCV/HBV/HCV+HBV, other) and screening status. A log transformation was made to AFP and bilirubin because of extreme skewness. To make allowance for lead-time bias introduced by systematic surveillance, we applied the method of Duffy *et al* (2008). Using forward selection, a multivariable Cox proportional hazards model was built to explain variation in survival as related to clinical features and aetiological factors.

## Results

**Figure 1:**
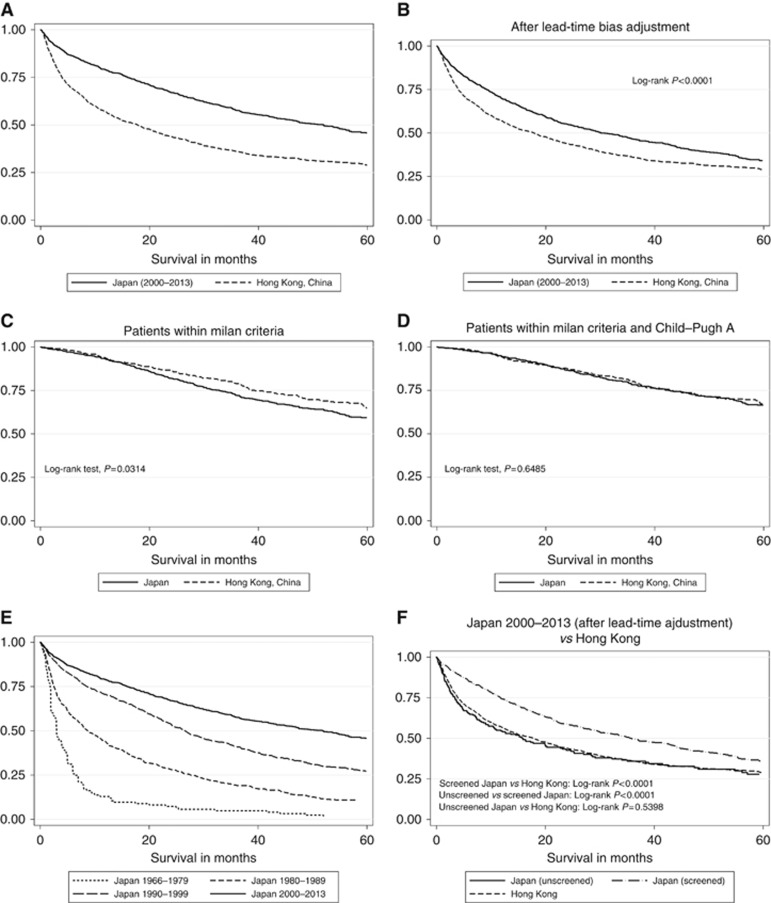
Kaplan–Meier curves showing survival. (**A**) In the Japanese and Chinese cohorts, (**B**) in the Japanese and Chinese cohorts after lead-time bias, (**C**) in Japanese and Chinese patients who were within the Milan criteria, (**D**) in Japanese and Chinese patients who were within the Milan Criteria and Child–Pugh A, (**E**) in Japan over the decades (1966–1979, 1980–1989, 1990–1999 and 2000–2013) and (**F**) survival according to screening status (after lead-time bias adjustment) in the Japanese (and Chinese) cohorts.

Comparing similar time periods (Japan 2000–2013; Hong Kong, China 2003–2014), median survival in Japan was 52 months compared with 17.8 months in Hong Kong (Figure 1A). This difference in survival was maintained even after allowing for lead-time bias (Figure 1B). By all measures of disease extent and stage (tumour size <3 cm, multifocality, vascular invasion as well as the Milan Criteria), the Japanese cohort had much earlier disease at diagnosis (Table 2). However, within that cohort of Hong Kong patients who were detected at an early stage (i.e., within the Milan Criteria), the median survival was actually significantly better than for the analogous Japanese group (Figure 1C), although among those with good liver function (Child–Pugh grade ‘A') survival figures were virtually identical (Figure 1D) as were results among those who underwent surgical resection or who were classified as receiving curative theory (data not shown). Comparing the Kaplan–Meier survival curves for late-stage patients with tumour sizes of over 5 cm (outside the Milan criteria) showed that there was no statistically significant difference (*P*=0.2068) between the Japanese and Chinese patients (Supplementary Figure 1).

### Changes in survival following introduction of surveillance programme in Japan

In the latest cohort (2000–2013), 78% of Japanese HCC cases were detected by surveillance. The high current median survival seen in Japan was preceded by increasing survival rates over several decades (Figure 1E). Thus, between the years of 1966 and 1980 when there was no surveillance programme in place, median survival in Japan was <3 months (Toyoda *et al*, 2006a). Survival improved over each following decade, from 8.8 months between 1980 and 1989 to the most recent figure of over 4 years (2000–2013; Figure 1E). The median age at diagnosis also increased each decade, from 60.5 years before the initiation of a surveillance programme to 70 years during the most recent analysis period (Table 3). In parallel with these changes, there was a shift towards earlier disease stage with the proportion of patients with stage 0/1 (the earliest stages according to the JIS), rising from 3.4% between 1966 and 1979 to 53.4% between 2000 and 2013 (Table 3).

In the Japanese data set, a clear distinction had been recorded between those detected within the formal surveillance programme and the remaining ‘unscreened' patients. Those who were screened had an earlier disease stage compared with those who were unscreened. For example, percentages for receiving curative treatment, within the Milan Criteria, tumour sizes <3 cm, multifocality and vascular invasion were 40.6%, 27.7%, 22.3%, 62.5% and 41.7%, respectively, for those who were unscreened compared with 69.1%, 71.8%, 61.5%, 38.4% and 8.7%, respectively, for screened patients. We therefore applied the previously referenced statistical method to assess the contribution of lead- and length-time bias to this cohort. This showed that the difference between the screened and unscreened cohorts decreased from 46.3 to 19.8 months but remained highly significant (*P*<0.0001; Figure 1F and Supplementary Figure 2). Median survival in other subgroups are summarised in Table 3.

### Role of aetiology and surveillance in HCC survival

Direct comparison between Japan and Hong Kong is complicated by major differences in aetiology, with Japanese patients being predominately HCV related and Hong Kong patients HBV related. Despite this in both aetiologies, patients in Japan clearly survived longer than those in Hong Kong (Supplementary Figures 3a and b) and multivariable analysis (Table 4) showed that tumour-related factors, such as vascular invasion, AFP and tumour size, but not aetiology, accounted for these differences (Supplementary Tables 1 and 2). When disease stage factors are accounted for in a multivariable Cox regression analysis, screening status variable becomes insignificant (*P*>0.05), indicating that any differences in survival between the two groups (screened and unscreened) is accounted for by disease stage. All univariable analysis is shown in Supplementary Tables 3 and 4.

## Discussion

The stage of HCC at presentation was the most important factor influencing survival. Patients with early-stage disease are more likely to receive potentially curative therapy and survive longer. When we applied a statistical method that adjusts for lead and length-time bias, significant benefit remained among the screened population in Japan (log-rank test, *P*<0.0001). This method has limitations in that it is not specific for HCC but other approaches that make allowance for lead-time bias, using HCC-specific features, have arrived at similar conclusions. Specifically Mourad *et al* (2014), found the same using a modelling approach and Cucchetti *et al* (2014) concluded that even after lead-time bias adjustment, semiannual surveillance maintained a survival benefit over symptomatic diagnosis. The fact that the median age at diagnosis in Japan has not fallen since the surveillance programme was initiated, but rather increased, may offer further evidence that lead-time bias does not account for all the benefit of surveillance. The better survival in Japan is unlikely to be attributable simply to ‘better' treatment as, among those Hong Kong patients detected within the Milan Criteria, the latter actually survived longer than the Japanese cohort and by all other measures of survival in early-stage disease according to treatment there were no significant differences.

The progressive improvement in median survival between 1980 (when surveillance was initiated) and 2013 (from <3 months to the current figure of >70 months) in the Japanese cohort has been replicated across Japan (Ikai *et al*, 2010). This observation cannot, in itself, be taken as evidence for the benefit of surveillance since there have, over the same period, been major advances in both diagnosis and management. For example, we cannot be entirely confident of the diagnosis of small HCCs in the early stages of the study, before internationally agreed diagnostic criteria were established. However, most small tumours did come to resection and were thus histologically confirmed. Crucially however, whilst in Hong Kong the survival has increased from 3 months (Shiu *et al*, 1990) to 17.8 months, in Japan the improvement (over the same time period) has been to 52 months (30 months after adjusting for lead-time bias). Furthermore, the parallel stage-shift to earlier disease (as assessed by the JIS system) supports the contention that survival improvement was, at least in part, attributable to surveillance. In both Hong Kong and Japan, patients with chronic HCV survived longer than those with HBV, suggesting that the high incidence of HCV infection might contribute to the better survival in Japan compared with Hong Kong. A direct comparison, however, reveals that within each aetiology, Japanese patients consistently survived longer. Interestingly, in both Japan and Hong Kong, irrespective of how the HCC cases were detected, those with HBV had clinical features characteristic of more advanced disease.

However, there are significant limitations to our study. Crucial issues such as cost effectiveness, and any harm inflicted by a surveillance programme such as the consequence of false-positive results, have not been considered. Furthermore, any benefit of surveillance suggested here is not necessarily transferable to a Western setting. Obesity is increasingly recognised as an aetiological factor for HCC development in the West and this will decrease the sensitivity of US examination, whereas US is likely to be a more effective surveillance tool in the slimmer Japanese population (Zaman, 2013). In Japan, patients at risk are a well-informed population committed to surveillance and this may not be the case in the West where compliance may be poor, especially among those with alcoholic cirrhosis. Furthermore, only patients in whom the presence of a risk factor for HCC is known (e.g., chronic viral hepatitis) will enter a surveillance programme. In Hong Kong, HCC was often the first manifestation of chronic hepatitis B infection, whereas in Japan the population had already been offered screening for the presence of chronic HBV or HCV. There is abundant evidence from the United States that management strategies developed and implemented in specialist centres are not always replicated in the primary care setting and that the overall percentage of patients with cirrhosis actually undergoing effective surveillance is very low (El-Serag and Davila, 2010; Dalton-Fitzgerald *et al*, 2014; Joshi *et al*, 2014). The multiple barriers that inhibit translation of the potential benefits of surveillance into an effective program at the population level have been clearly described by Singal and El-Serag (2015).

Although a prospective randomised controlled trial (RCT) to assess the impact of surveillance would be ideal, it is now recognised that this approach is not practical (Poustchi *et al*, 2011; McCaughan, 2013; Kansagara *et al*, 2014; Singal *et al*, 2014; Sherman, 2014a, 2014b); all other, non-RCT-based approaches have significant limitations. Nonetheless, in the absence of an RCT, our data when combined with the time trends shown here, and reports from the whole of Japan (Toyoda *et al*, 2006a) and other parts of Asia (Yeh *et al*, 2014), lend strong support for the beneficial impact of surveillance on HCC mortality.

## Figures and Tables

**Table 1:** Characteristics of the cohorts

	**Japan**		**Hong Kong, China**
***N***	**2605**		**1675**
Accrual period	1966–1999, *n*=1431	2000–2013, *n*=1174	2003–2014
% Ethnicity	>95% (Oriental)	>95% (Oriental)	>95% (Oriental)
Age (years)			
Median (IQR)	63 (56, 69)	70 (63, 76)	59 (52, 68)
Mean (±s.d.)	62.6 (±9.5)	68.8 (±9.5)	59.6 (±11.4)
% Male	75.8, *n*=1431	70.8, *n*=1174	84.6, *n*=1675
% Aetiology	*n*=1326	*n*=1174	*n*=1112
HCV	48.8	66.4	6.7
HBV	21.6	15.7	80.5
HCV+HBV	0.9	0.9	0.5
Other^a^	28.7	17	12.2
**Liver function and cancer biomarkers**			
AFP (ng ml^−1^), median (IQR)	68.0 (14, 1130), *n*=1216	21.3 (6.3, 208.5), *n*=1156	107 (9, 2869), *n*=1675
Bilirubin (*μ*mol l^−1^), median (IQR)	17.1 (10.3, 29.1), *n*=1404	13.7 (10.3, 22.2), *n*=1168	15 (10, 26), *n*=1675
Albumin (g l^−1^), median (IQR)	32 (27, 36), *n*=1375	36 (32, 40), *n*=1168	38 (34, 42), *n*=1675
% Child score (A : B : C)	46.0 : 39.1 : 15.0, *n*=1429	70.2 : 22.5 : 7.3, *n*=1174	75.9 : 20.2 : 3.9, *n*=1675
**Tumour characteristics and disease stage**			
% Multifocal	67.4, *n*=1431	43.8, *n*=1165	45.4, *n*=1675
Tumour size	*n*=1043	*n*=1164	*n*=1600
<3 cm (%)	44.9	52.8	25.3
3–5 cm (%)	27.1	22.3	25.6
5.1–10 cm (%)	20.9	18.9	25.9
>10 cm (%)	7.1	6	23.2
% Vascular invasion (presence)	42.4, *n*=1341	16, *n*=1163	26.7, *n*=1675
% HCC detected through surveillance	59.50, *n*=1312	77.60, *n*=1172	NA
% within the Milan Criteria	35.2, *n*=1430	62, *n*=1159	31.7, *n*=1624
Treatment (% curative)	29.3, *n*=1431	62.8, *n*=1170	44.1, *n*=1675
**Survival**			
Median overall survival (months)	16.6, *n*=1430	52, *n*=1174	17.8, *n*=1672

Abbreviations: AFP=*α*-fetoprotein; HBV=hepatitis B virus; HCC=hepatocellular carcinoma; HCV=hepatitis C virus; IQR=interquartile range; NA=not applicable.

a Other aetiology includes alcoholic, fatty liver disease, haemochromatosis, autoimmune hepatitis and cryptogenic.

**Table 2:** Percentage of patients with curative treatments, early-stage BCLC and within the Milan Criteria

**Cohort**	**% screened**	**% curative**	**% within Milan Criteria**	**% tumour size <3 cm**	**% multifocal**	**% vascular invasion**
Japan 1966–1979	12.9 (*n*=132)	3.3 (*n*=150)	6.0 (*n*=149)	12.5 (*n*=16)	93.3 (*n*=150)	86.3 (*n*=73)
Japan 1980–1989	53.1 (n=375)	16.2 (*n*=476)	20.0 (*n*=476)	30.2 (*n*=291)	75.2 (*n*=476)	62.9 (*n*=464)
Japan 1990–1999	70.1 (*n*=805)	41.9 (*n*=805)	50.1 (*n*=805)	51.4 (*n*=736)	57.9 (*n*=805)	26.6 (*n*=804)
Japan 2000–2013	77.6 (*n*=1172)	62.8* (*n*=1170)	62.0* (*n*=1159)	52.8* (*n*=1164)	43.8** (*n*=1165)	16.0* (*n*=1163)
Hong Kong	NA	44.1* (*n*=1675)	31.7* (*n*=1624)	25.3* (*n*=1600)	45.4** (*n*=1675)	26.7* (*n*=1675)

Abbreviation: BCLC=Barcelona-Clinic Liver Cancer.

Note: Comparing Japan (2000–2013) and Hong Kong, China. *P<0.0001 and **P=0.4.

**Table 3:** Comparing recent Japanese cohort with those over the decades as well as Hong Kong, China cohort

	**Median survival, in months (95% CI)**										
**Data set**	**Overall**^**†**^ **Figure 1A and E**	**Overall (after lead-time adjustment)**^**†**^ **Figure 1B**	**Within Milan Criteria**^**†**^ **Figure 1C**	**within Milan Criteria and Child-Pugh A† Figure 1D**	**Unscreened Japan**^**†**^ **Figure 1F**	**Screened Japan**^**†**^ **Figure 1F**	**% resections**	**Crude 90-day mortality rate for resections (per 1000)**	**Median tumour size (cm)**	**JIS early stage, % (stage 0/1)**	**Median age (years)**
Japan 1990–1999	26.6 (24.1, 29.2), *n*=805	NA	NA	NA	NA	NA	17.0, *n*=805	21.9	2.8, *n*=736	38.7, *n*=805	64.0, *n*=805
Japa*n* 1980–1989	8.8 (7.2, 11.1), *n*=475	*N*A	*N*A	NA	NA	NA	12.4, *n*=475	101.7	3.9, *n*=291	12.9, *n*=475	60.0, *n*=475
Japan 1966–1979	3.0 (2.6, 3.9), *n*=150	NA	NA	NA	NA	NA	3.3, *n*=150	0	3.5, *n*=16	3.4, *n*=150	60.5, *n*=150
Japan 2000–2013	52.0 (44.1, 57.0), *n*=1174	30.0 (25.9–35.8), *n*=1174	77.3 (67.8, 88.2) *n*=718	95.6 (78.1, 114.8) *n*=559	15.9 (10.5, 23.2) *n*=263	35.7 (30.0, 42.9) *n*=909	37.5*, *n*=1174	22.7**	2.8*, *n*=1164	53.4, *n*=1174	70.0*, *n*=1174
Hong Kong, China	17.8 (15.0, 20.2), *n*=1672	NA	97.6 (82.2,), *n*=515	103.1 (87.5,), *n*=489	NA	NA	27.6*, *n*=1672	13**	5.0*, *n*=1600	NA	59.0*, *n*=1672

Abbreviations: CI=confidence interval; NA=not applicable.

Note: See figures for the log-rank tests^†^. Comparing Japan (2000–2013) and Hong Kong, China. *P<0.0001, *t*-test and **P=0.1014.

**Table 4:** Multivariable Cox regression analysis

_***t***	**Haz. ratio**	**s.e**	***Z***	***P*****>*****z***	**95% Conf. interval**	
**Japan**						
Vascular invasion						
No	Ref.					
Yes	2.815	0.397	7.34	<0.0001	2.136	3.71
Albumin	0.91	0.008	−11.16	<0.0001	0.895	0.925
Log 10 AFP	1.279	0.047	6.75	<0.0001	1.191	1.373
Age	1.034	0.005	6.25	<0.0001	1.023	1.045
Tumour type						
Solitary	Ref.					
Multifocal	1.605	0.153	4.97	<0.0001	1.332	1.935
Tumour size	1.05	0.008	6.22	<0.0001	1.034	1.067
Log 10 bilirubin	2.226	0.407	4.38	<0.0001	1.556	3.186
Gender						
Female	Ref.					
Male	1.519	0.152	4.18	<0.0001	1.249	1.849
**Hong Kong, China**						
Vascular invasion						
No	Ref.					
Yes	2.501	0.186	12.35	<0.0001	2.162	2.892
Log 10 bilirubin	2.625	0.263	9.65	<0.0001	2.158	3.194
Tumour size	1.069	0.007	9.64	<0.0001	1.054	1.083
Albumin	0.949	0.006	−8.76	<0.0001	0.938	0.96
Log 10 AFP	1.177	0.027	7.2	<0.0001	1.126	1.231
Tumour type						
Solitary	Ref.					
Multifocal	1.566	0.106	6.64	<0.0001	1.372	1.788
Age	1.008	0.003	2.62	0.009	1.002	1.013

Abbreviations: AFP=*α*-fetoprotein; Ref.=reference.
