# Supplementary material for: Impact of disease stage and aetiology on survival in hepatocellular carcinoma: implications for surveillance
Source: Br J Cancer. 2017 Jan 12;116(4):441–7. doi: 10.1038/bjc.2016.422 (PMC5318967; doi:10.1038/bjc.2016.422)
Supplement: Supplementary Information [file bjc2016422x1.docx]

**SUPPLEMENTARY DATA**

| **Supplementary Table 1: Summary of multivariable Cox regression** | | |
| --- | --- | --- |
| **Variables** | **Japan** | **Hong Kong, China** |
| **Age** | √ | √ |
| **Gender (male)** | √ |  |
| **Log10 bilirubin** | √ | √ |
| **Albumin** | √ | √ |
| **Log10 AFP** | √ | √ |
| **Tumour size** | √ | √ |
| **Tumour type (multifocal)** | √ | √ |
| **Vascular Invasion** | √ | √ |
| **Aetiology** | **×** | **×** |

| **Supplementary table 2 – Comparison of prognostic factors between HCV and HBV positive patients in Japan and Hong Kong, China.** | | | |
| --- | --- | --- | --- |
|  | | **Japan (2000 – 2013)** | **China** |
| **HCV** | **Age (median, IQR and mean ±SD)** | 71 (66 – 76)  70.4 (± 8.2)  n=780 | 63 (57 – 71)  63.6 (±10.0)  n=112 |
|  | **AFP (median, IQR )** | 21.4 (7.2 – 137.7)  n=770 | 80.5 (17 – 555.5)  n=112 |
|  | **Tumour size (median, IQR )** | 2.5 (1.7 – 4.2)  n=775 | 3.2 (2.5 – 5)  n=105 |
|  | **Within Milan Criteria %** | 68.1  n=771 | 47.6  n=103 |
|  | **Solitary tumours %** | 57.7  n=776 | 61.6  n=112 |
|  | **Vascular invasion %** | 12.3  n=774 | 14.3  n=112 |
| **HBV** | **Age (median, IQR and mean ±SD)** | 59 (54 – 66)  59.3 (±10.7)  n=184 | 58 (51 – 66)  58.3 (± 11.0)  n=1349 |
|  | **AFP (median, IQR )** | 33.6 (5.6 – 750.3)  n=178 | 129 (10.0 –3601)  n=1349 |
|  | **Tumour size (median, IQR )** | 3.1 (1.8 – 7.3)  n=180 | 5.0 (2.9 – 9.7)  n=1293 |
|  | **Within Milan Criteria %** | 48.6  n=181 | 32.3  n=1311 |
|  | **Solitary tumours %** | 51.9  n=181 | 54.3  n=1349 |
|  | **Vascular invasion %** | 28.7  n=181 | 26.9  n=1349 |

* Sant M, Allemani C, Santaquilani M, et al. European Journal of Cancer. 2009;45(6):931-91

† http://www.ons.gov.uk/ons/publications/re-reference-tables.html?edition=tcm%3A77-252716.

^ http://www.cancer.org/research/cancerfactsfigures/globalcancerfactsfigures/global-facts-figures-2nd-ed.

× http://seer.cancer.gov/csr/1975_2011/sections.html.

# ACS. Cancer Facts & Figures 2014 - American Cancer Society.

**Toyoda H, Kumada T, Osaki Y, et al. Clinical Gastroenterology and Hepatology. 2006;4(12):1528-36.

´´Kudo M, Chung H, Osaki Y. Journal of gastroenterology. 2003;38(3):207-15.

| **Supplementary Table 3: Univariable Cox regression analysis – Japan (2000 – 2013)** | | | | | | |
| --- | --- | --- | --- | --- | --- | --- |
| **Variable** | **Haz. Ratio** | **Std. Err.** | **z** | **P>z** | **[95% Conf. Interval]** | |
| **Age** | 1.024 | 0.005 | 4.68 | <0.0001 | 1.014 | 1.034 |
| **Gender (male)** | 1.066 | 0.0997 | 0.68 | 0.497 | 0.887 | 1.280 |
| **Log10 bilirubin** | 7.755 | 1.302 | 12.20 | <0.0001 | 5.581 | 10.777 |
| **Albumin** | 0.894 | 0.006 | -15.64 | <0.0001 | 0.882 | 0.907 |
| **Log10 AFP** | 1.575 | 0.0525 | 13.62 | <0.0001 | 1.475 | 1.681 |
| **Treatment (palliative)** | 4.542 | 0.398 | 17.25 | <0.0001 | 3.825 | 5.394 |
| **Tumour size** | 1.074 | 0.005 | 16.79 | <0.0001 | 1.065 | 1.083 |
| **Tumour type (multifocal)** | 2.620 | 0.225 | 11.20 | <0.0001 | 2.213 | 3.100 |
| **Vascular Invasion** | 6.156 | 0.645 | 17.34 | <0.0001 | 5.013 | 7.560 |
| **Aetiology** |  |  |  |  |  |  |
| **HCV** | 1 | - | - | - | - | - |
| **HBV** | 0.823 | 0.102 | -1.57 | 0.117 | 0.646 | 1.050 |
| **HCV+HBV** | 0.940 | 0.424 | -0.14 | 0.891 | 0.388 | 2.274 |
| **Other** | 1.229 | 0.144 | 1.76 | 0.079 | 0.976 | 1.546 |

| **Supplementary Table 4: Univariable Cox regression analysis – Hong Kong, China** | | | | | | |
| --- | --- | --- | --- | --- | --- | --- |
| **Variable** | **Haz. Ratio** | **Std. Err.** | **z** | **P>z** | **[95% Conf. Interval]** | |
| **Age** | 1.006 | 0.003 | 2.01 | 0.045 | 1.000 | 1.011 |
| **Gender (male)** | 1.142 | 0.098 | 1.55 | 0.121 | 0.965 | 1.352 |
| **Log10 bilirubin** | 5.765 | 0.460 | 21.97 | 0.000 | 4.931 | 6.740 |
| **Albumin** | 0.905 | 0.004 | -20.11 | <0.0001 | 0.896 | 0.914 |
| **Log10 AFP** | 1.477 | 0.030 | 19.34 | <0.0001 | 1.420 | 1.537 |
| **Treatment (palliative)** | 7.936 | 0.623 | 26.37 | <0.0001 | 6.804 | 9.257 |
| **Tumour size** | 1.128 | 0.007 | 20.68 | <0.0001 | 1.115 | 1.141 |
| **Tumour type (multifocal)** | 2.556 | 0.161 | 14.94 | <0.0001 | 2.260 | 2.891 |
| **Vascular Invasion** | 4.557 | 0.301 | 22.98 | <0.0001 | 4.004 | 5.187 |
| **Aetiology** |  |  |  |  |  |  |
| **HCV** | 1 | - | - | - | - | - |
| **HBV** | 1.084 | 0.134 | 0.65 | 0.513 | 0.851 | 1.382 |
| **HCV+HBV** | 3.810 | 1.352 | 3.77 | 0.000 | 1.900 | 7.637 |
| **Other** | 1.258 | 0.183 | 1.58 | 0.115 | 0.946 | 1.674 |

**Supplementary Figure 1**

| **Country** | **N** | **Median survival in months (95% C.I.)** |
| --- | --- | --- |
| **Japan** | 80 | 5.6 (2.7 – 8.3) |
| **Hong Kong, China** | 445 | 3.9 (3.4 – 4.5) |

**Supplementary Figure 2**

| **Status** | **N** | **Median survival in months, before lead-time adjustment (95% C.I.)** |
| --- | --- | --- |
| **Unscreened** | 263 | 15.9 (10.5 – 23.2) |
| **Screened** | 909 | 62.2 (53.7 – 67.8) |

**Supplementary figure 3**

**(b)**

**(a)**

| **Country** | **N** | **Median survival in months (95% C.I.)** |
| --- | --- | --- |
| **Japan** | 780 | 51.7 (43.3 – 56.6) |
| **Hong Kong, China** | 112 | 27.2 (17.7 – 35.5) |

| **Country** | **N** | **Median survival in months (95% C.I.)** |
| --- | --- | --- |
| **Japan** | 184 | 80.1 (44.1 – 135.9) |
| **Hong Kong, China** | 1346 | 17.9 (14.9 – 20.5) |

**Supplementary Figure Legends**

**Supplementary Figure 1:** Kaplan-Meier curves showing survival according to Japanese and Chinese cohorts in patients with tumour sizes greater than 5cm (outside Milan criteria).

**Supplementary Figure 2:** Kaplan-Meier curves showing survival according to screening status (prior to lead-time bias adjustment) in the Japanese cohort.

**Supplementary figure 3:** Kaplan-Meier curves showing survival according to (a) HCV and (b) HBV in the Japanese and Chinese cohorts.
