# Supplementary figures and images for: Impact of disease stage and aetiology on survival in hepatocellular carcinoma: implications for surveillance
Source: Br J Cancer. 2017 Jan 12;116(4):441–7. doi: 10.1038/bjc.2016.422 (PMC5318967; doi:10.1038/bjc.2016.422)

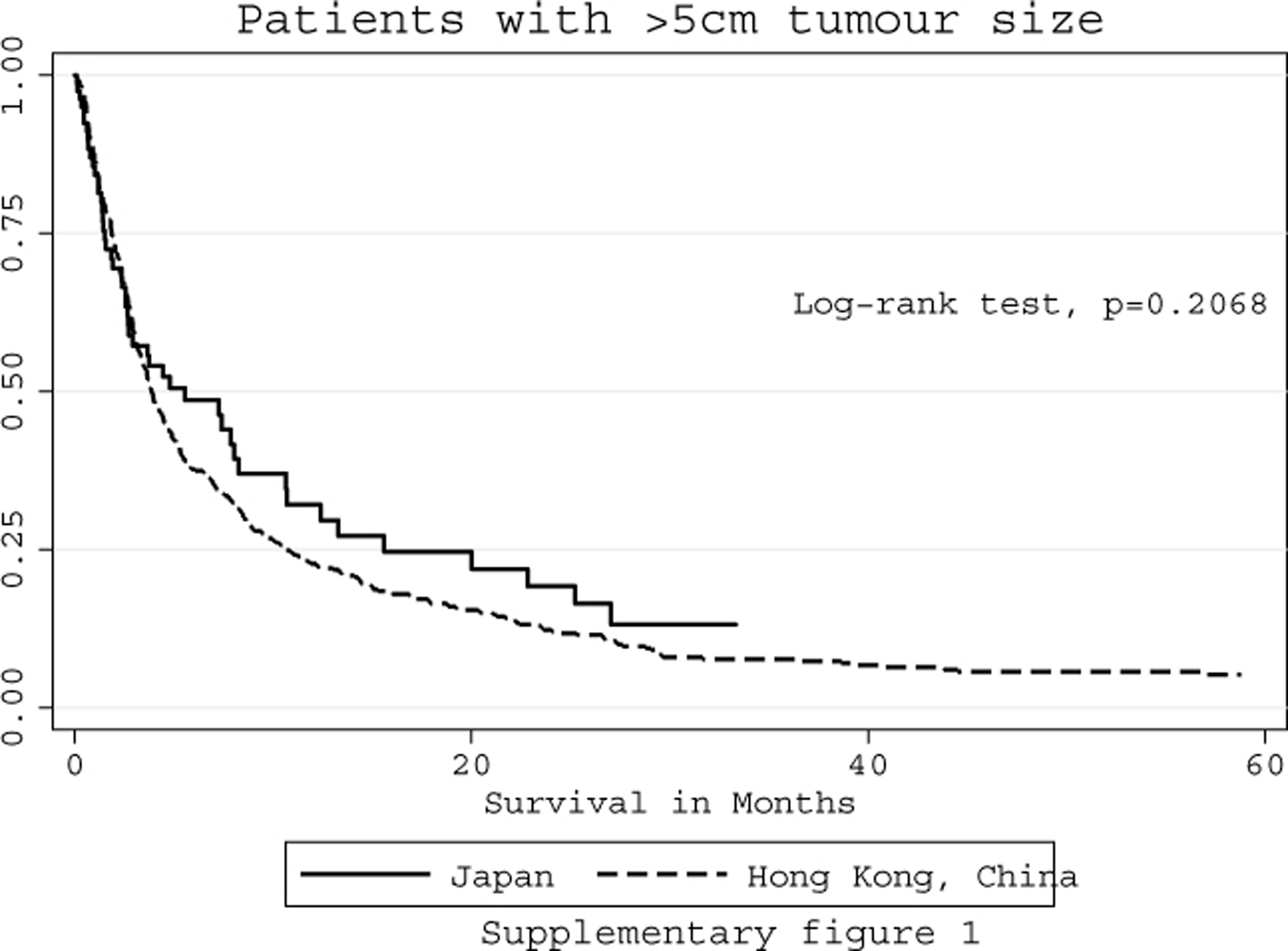

Supplement: Supplementary Figure S1 [file bjc2016422x2.tif]

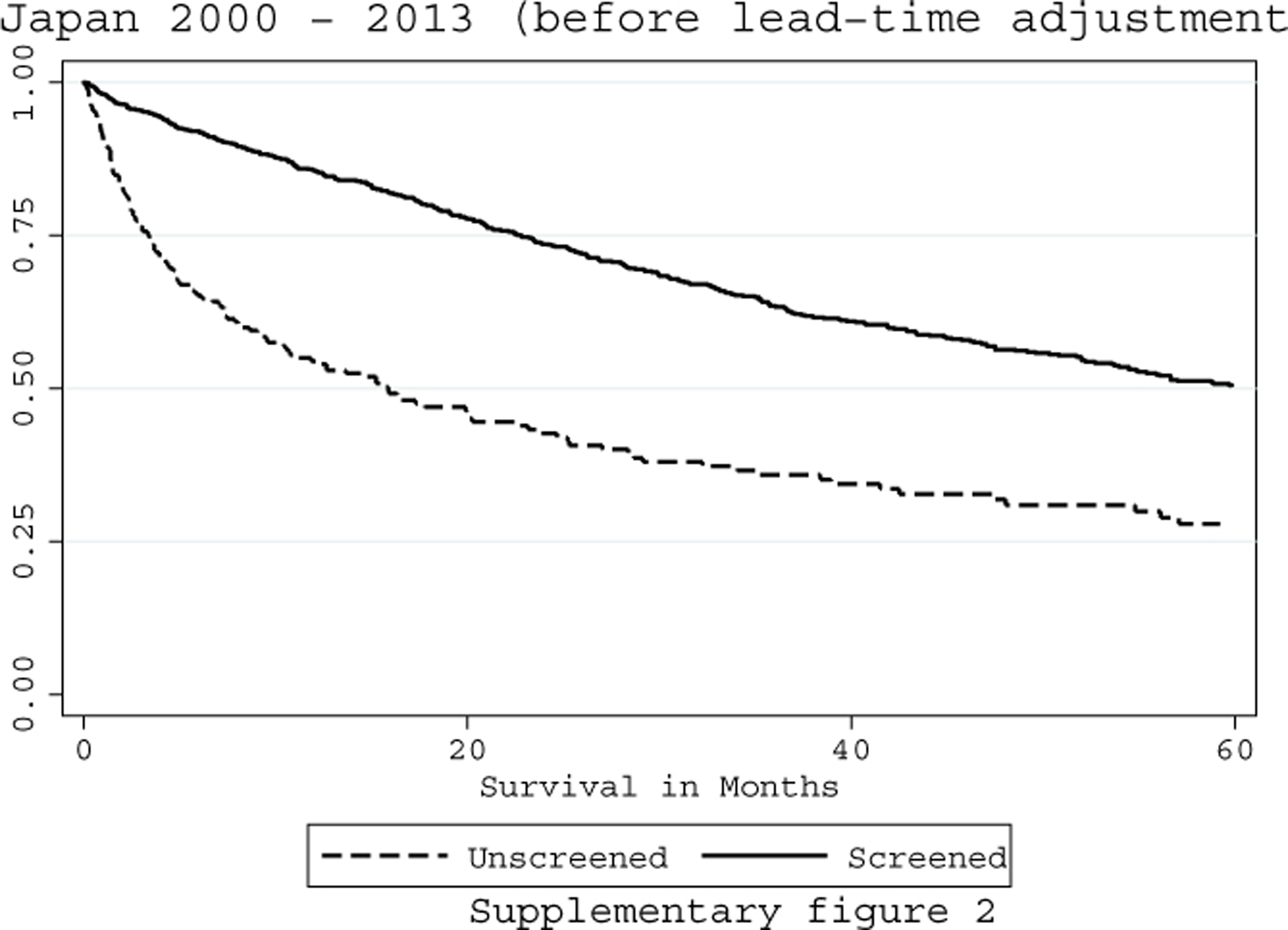

Supplement: Supplementary Figure S2 [file bjc2016422x3.tif]

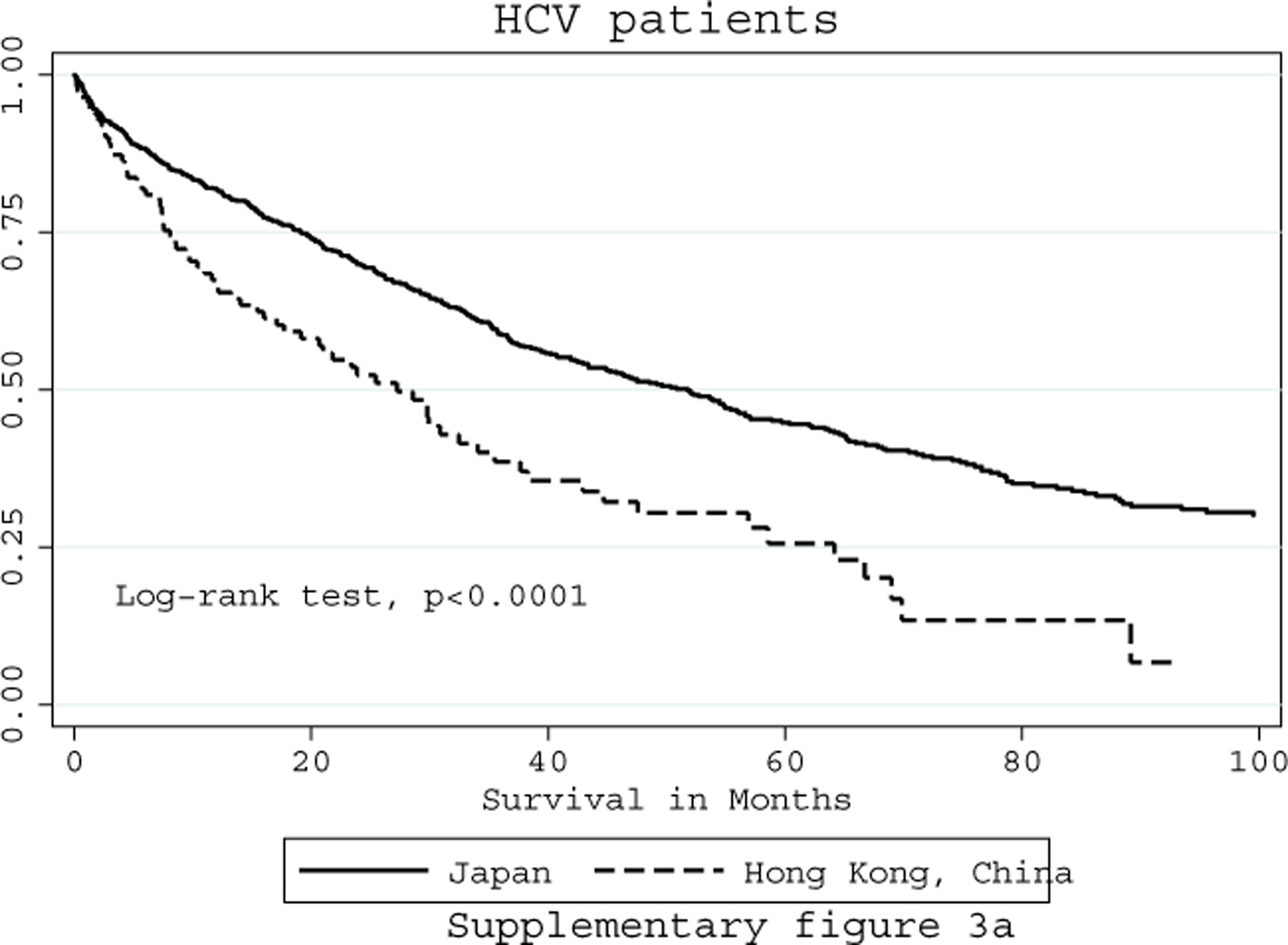

Supplement: Supplementary Figure 3a [file bjc2016422x4.tif]

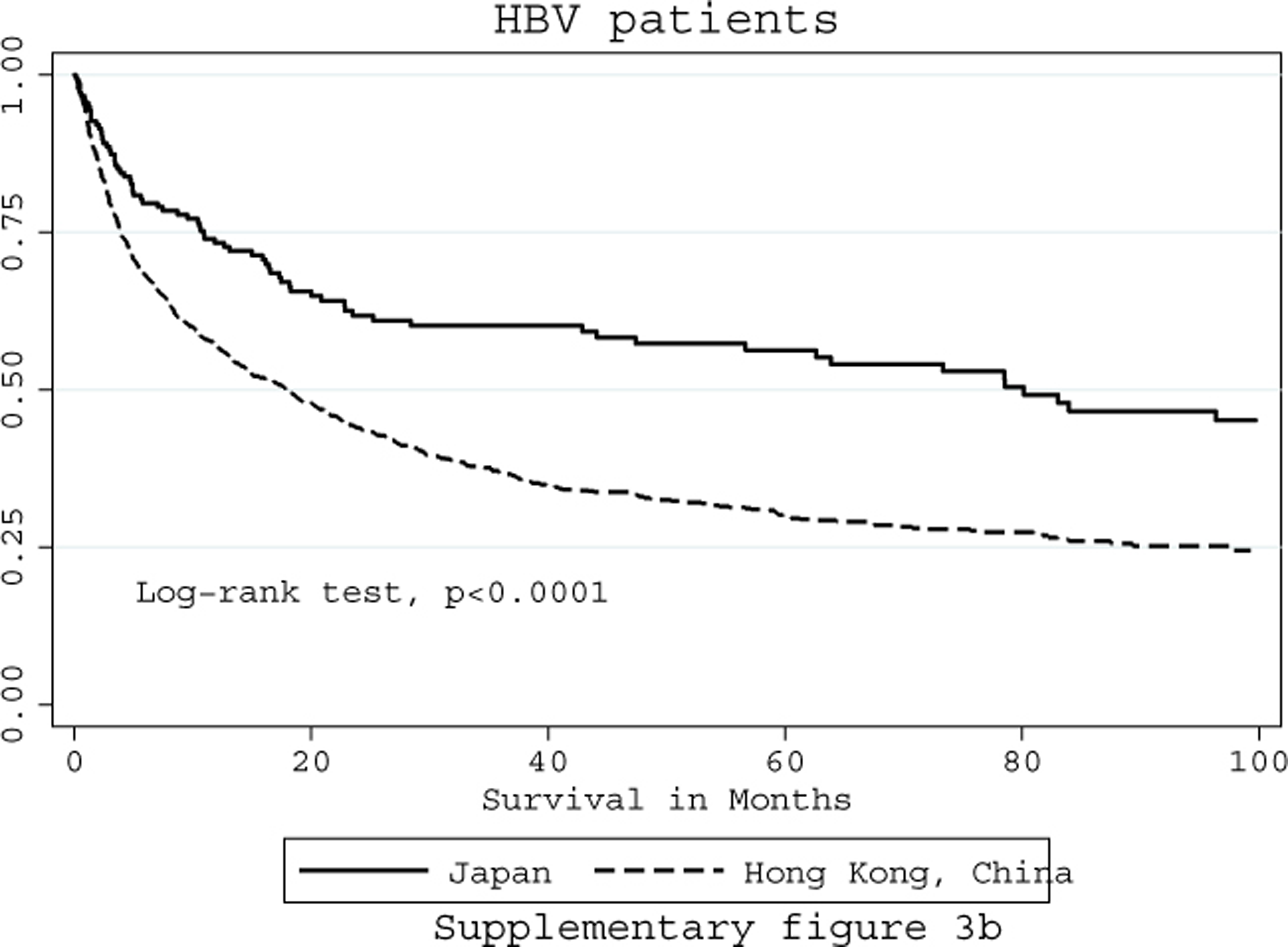

Supplement: Supplementary Figure 3b [file bjc2016422x5.tif]
